# Secretory Production of Heterologous Antimicrobial Peptides in *Corynebacterium glutamicum*

**DOI:** 10.1002/elsc.70008

**Published:** 2025-02-18

**Authors:** Wei Long, Lina Apitius, Patrick Lenz, Felix Jakob, Anna Joёlle Ruff, Ulrich Schwaneberg

**Affiliations:** ^1^ Lehrstuhl für Biotechnologie RWTH Aachen University Aachen Germany; ^2^ Bioeconomy Science Center (BioSC) c/o Research Center Jülich Jülich Germany; ^3^ DWI – Leibniz‐Institut für Interaktive Materialien Aachen Germany

**Keywords:** antimicrobial peptides, *Corynebacterium glutamicum*, expression, production, secretion

## Abstract

Antimicrobial peptides (AMPs) are host defense peptides that act against a broad spectrum of microorganisms. AMPs are of high interest as medicinal products, antimicrobial coatings, and for controlling biofilm formation. Applications and research of many AMPs are still hampered by insufficient titers and lack of production platforms that can tolerate high titers of AMPs. *Corynebacterium glutamicum* is an excellent microbial host for protein secretion and has been barely explored as a host for AMP production. Here, we report the successful production and secretion of two AMPs (amounts of up to 130 mg/L for liquid chromatography peak I [LCI] and 54 mg/L for Psoriasin) by *C. glutamicum* with low amounts of secreted byproducts.

**Abbreviations**
AMP: antimicrobial peptide
LCI: liquid chromatography peak I

## 1 Short Communication

Antimicrobial peptides (AMPs) are small peptides (up to 100 amino acids) with a large fraction of hydrophobic moieties and broad antimicrobial activity against microorganisms [1]. They are naturally produced by living organisms as part of their innate immune system [2]. Applications include therapeutic usage [3] for example against infections [4] as viable alternatives to conventional antibiotics [5], functionalization of polymers such as antimicrobial surface coatings [6, 7] and for limiting biofilm formation [8]. A major advantage compared to common antibiotics is the improbable development of resistance by microorganisms against AMPs [8, 9]. The availability of the peptides in sufficient quantities is often a limiting factor for researching their characteristics and usage [10, 11]. AMPs can be gained from their natural producer, which often results in low yields [11, 12] or through chemical synthesis, which is competitive for small peptides but not for the production of AMPs longer than 40 aa [12, 13, 14]. Recombinant production in microbial hosts like *Escherichia coli* and *Bacillus subtilis* and yeasts like *Pichia pastoris* is an attractive alternative for AMP production [15] with titers up to 2.7 g/L [16]. Challenges in recombinant AMP expression include toxic effects on the host due to antimicrobial activity, aggregation resulting from low solubility, low expression, and proteolytic degradation by endogenous proteases. Common successful strategies to optimize peptide production are production as fusion proteins [17] or multimers in tandem repeats [18], co‐expression of toxicity quenchers [19], and production in inclusion bodies [20].

**Summary**

- The rise of antibiotic‐resistant pathogenic bacteria has highlighted antimicrobial peptides (AMPs) as important and promising candidates for new antibiotic therapies.
- The successful production and secretion of AMPs by *Corynebacterium glutamicum* presents significant practical applications.
- With the ability to produce liquid chromatography peak I (LCI) and Psoriasin at titers of 130 and 54 mg/L, respectively, this research offers a high‐purity alternative to traditional recombinant production hosts like *E. coli* and yeast.
- The low levels of secreted byproducts facilitate simple and cost‐effective AMP isolation, which is crucial for developing medicinal products, antimicrobial coatings, and biofilm control strategies.
- The direct immobilization of these secreted peptides in 96‐well microtiter plates allows for potential applications in directed enzyme evolution and streamlined cell removal.
- Overall, this work underscores the potential of *C. glutamicum* as a robust platform for large‐scale AMP production, opening new avenues for research and product development in antimicrobial therapies.

Heterologous production of secreted AMPs is a favorable production process that allows cost‐effective downstream processing, reduced proteolytic degradation, and increased production titers. AMP fusion to signal peptides or to secreted fusion partners can promote secretion [21, 22, 23]. Successful examples were the production of a plectasin variant in *P. pastoris* with amounts up to 1.3 g/L [24] and secretory production of the AMP bombinin in *E. coli* as a fusion construct with amounts of up to 300 mg/L [22]. Despite numerous successful examples, expression is still limiting for many AMPs. The Gram‐positive bacterium *Corynebacterium glutamicum* is a promising candidate for secretory production due to its low extracellular proteolytic activity [25] and small amounts of extracellular proteins [26]. *C. glutamicum* is an established industrial production host for amino acids [27] and has been used for the successful expression and secretion of several heterologous proteins, including proteases, nucleases, endoxylanases, and amylases [25, 28, 29]. *C. glutamicum* is known to produce its own AMPs [30] and was additionally utilized for the production of recombinant AMPs such as pediocin, nisin, and garvicin Q [31, 32, 33].

In this work, we investigated the strain *C. glutamicum* ATCC 13032 as a production host for the secretion of the AMPs LCI and Psoriasin. Liquid chromatography peak I (LCI) is a peptide from *B. subtilis*, which consists of 47 aa forming a four‐stranded antiparallel β‐sheet [34]. The antimicrobial activity of LCI was proven against *Xanthomonas campestris* pv Oryea und *Pseudomonas solanacearum* PE1 [35]. LCI is a preferred polymer binding peptide [6] and was subjected to directed evolution to improve binding strength and detergent tolerance [36, 37]. Psoriasin is an 11 kDa, secreted peptide gained from human skin cells [38, 39]. It can be found in high concentrations in wounds and plays a role in killing *E. coli* cells [40].

We employed the *C. glutamicum–E. coli* shuttle vector pEKEx2 with the signal peptide NprE [41] to produce secreted AMP in *C. glutamicum*. The published fusion construct eGFP‐17xHelix‐TEV‐LCI for the production of LCI in *E. coli* by Rübsam et al. [6, 37] was used as a template for the construct design (Figure S1, Table S1). The reported fusion proteins consisted of an AMP domain, which was linked via a 17 aa spacer [42] and a TEV cleavage site [43] to a fluorescent protein (eGFP). Instead of eGFP, we used the Strep‐tag II [44] which enabled a simple visualization in solution via 3,3′,5,5′‐tetramethylbenzidine (TMB) and was inserted between NprE and the 17xHelix spacer. The TEV cleavage site in front of the AMP allowed the cleavage of the N‐terminal tail and the production of native AMPs with one added glycine which remains after TEV cleavage. The phosphorothioate‐based ligase‐independent gene cloning (PLICing) [45] was chosen for the generation of AMP constructs using phosphorothioated primers (Table S1) which introduced the Strep‐taq II amino acid sequence.

**FIGURE 1:**
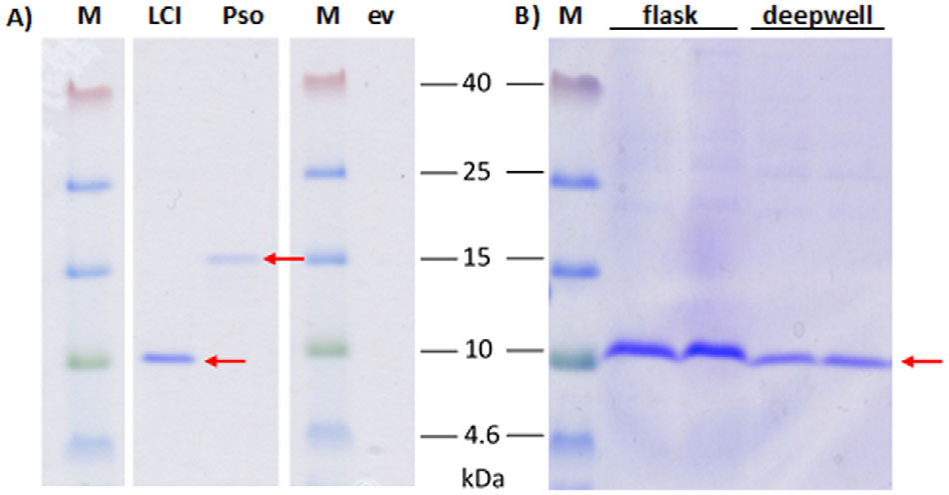
AMP expression in *C. glutamicum*. (A) LCI (9.4 kDa), Psoriasin (Pso) (15.4 kDa), and an empty vector control (ev) were expressed overnight in the flask (LB‐medium, 30°C, 250 rpm). (B) Optimized LCI production in flask and deepwell plate were visualized by Coomassie staining. Bands are indicated by red arrows and molecular weight was estimated by Spectra Multicolor Low Range Protein Ladder (Thermo Fischer Scientific, indicated with M). For expression conditions, Western blot analysis and quantification by ImageJ see Figures S2 and S3. AMP, antimicrobial peptide; LCI, liquid chromatography peak I.

Expression supernatant containing the secreted AMPs was analyzed by Tricine‐sodium dodecyl sulfate (SDS)‐gel electrophoresis (Figure 1) and Western blot without any concentration step. The Tricine‐SDS‐gel displayed a distinct band at 10 and 15 kDa which corresponded to the expected size of the constructs Strep‐tag II‐17xHelix‐TEV‐LCI (9.4 kDa) and Strep‐tag II‐17xHelix‐TEV‐Psoriasin (15.4 kDa). The more sensitive and specific detection via Western blot with Strep‐Tactin‐horseradish peroxidase (HRP) catalyzed oxidation of diaminobenzidine confirmed the expression of LCI and Psoriasin (Figure S3). *C. glutamicum* was, as expression host, able to produce LCI and Psoriasin without any optimization. Furthermore, a high level of purity of the secreted AMPs (LCI and Psoriasin) in the supernatant can be observed on the gel.

For flask production (25°C, 250 rpm), a 40 h expression in brain heart infusion (BHI) medium resulted in the highest titer (130 mg/L; determined with a Tricine‐SDS‐gel; see Figure 1). This corresponds to a yield of 14 µM LCI. Whilst, eGFP‐LCI production in *E. coli* as described by Rübsam et al. [6] yielded only 3.7 times lower yields (3.8 µM LCI). Production in deepwell plates resulted in LCI titer of 30 mg/L (Figures 1 and S2). Psoriasin production in the flask (LB, overnight, 30°C, 250 rpm) was quantified to reach 54 mg/L (Experion). In an additional investigation of an inhibitory effect of the AMPs on the expression host, only marginal growth inhibition could be detected at an AMP concentration 5‐fold higher than in the expression culture. Lower AMP concentrations did not show any changes in cell growth (Figure S4). This again underlines the usefulness of the production of these AMPs in *C. glutamicum*.

The observed high level of purity of AMPs in the supernatant of *C. glutamicum* (Figures 1 and S2) was an attractive feature since produced AMPs could, for instance, be used in binding experiments without further purification. Other recombinant secretion hosts such as *Bacillus* have significantly higher amounts of by‐products and proteolytic activities in the supernatant, as they have a highly developed proteolytic network [46].

**FIGURE 2:**
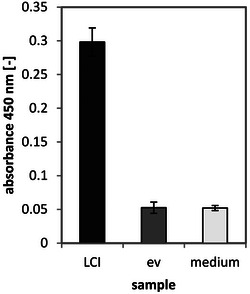
Immobilization of LCI on PS‐MTP surface. LCI was produced overnight in deepwell plates (LB‐medium, 900 rpm, 25°C, 70% humidity). The supernatant was obtained by centrifugation (4°C, 3220 × *g*, 20 min), diluted in PBS buffer (1:100), and loaded onto PS‐MTP (100 µL, 10 min, RT). The binding of produced LCI (dark) was determined with streptactin‐HRP conjugates (42 µg/L in PBS, 1 h, 600 rpm) and 1‐Step Ultra TMB ELISA solution (Thermo Fischer Scientific, Waltham, USA). The reaction was stopped with 2 M sulfuric acid and measured (450 nm, Tecan sunrise; TECAN, Männedorf, Switzerland). As controls, the absorbance of the empty vector (ev, gray) and the LB medium without cells (medium, light) are shown. The absorbance was calculated as the average of six measurements. HRP, horseradish peroxidase; LCI, liquid chromatography peak I; PS‐MTP, polystyrene‐microtiter plate.

The binding properties of produced AMPs to polystyrene‐microtiter plates (PS‐MTPs) [37] were determined with an HRP detection system [47] (Figure 2). The immobilized AMPs, which contained Strep‐tag II, were detected with a Strep‐Tactin‐HRP conjugate. HRP reduced hydrogen peroxide using TMB, which led to a colorimetric reaction [48]. The measured absorbance at 450 nm correlated to the amount of immobilized peptides/Strep‐Tactin‐HRP conjugates.

The results of the binding test showed that the LCI (Figure 2) and Psoriasin (data not shown) in the supernatant successfully bound directly to PS‐MTP surfaces.

In conclusion, we showed that *C. glutamicum* is a suitable host for efficient production of AMPs and a promising alternative to common production hosts such as *E. coli* and yeast. Two AMPs were directly secreted into the media and were expressed at high AMP levels. In case of LCI, a 3.7‐fold increase in titer was achieved compared to the expression in *E. coli*. A further attractive feature of *C. glutamicum* is the high purity of the secreted AMPs, which enables simple and cost‐effective isolation. Secreted LCI and Psoriasin enable direct peptide immobilization in 96‐well MTPs. The latter likely opens up novel applications for the secretory host *C. glutamicum*, for instance in directed enzyme evolution through secretion of LCI‐/Psoriasin‐fusion proteins, simple removal of cells, and quantification of the activity of immobilized enzyme variants in 96‐well MTPs.

## Conflicts of Interest

The authors declare no conflicts of interest.

## Ethics Statement

This article does not contain any studies with human participants or animals performed by any of the authors.

## Supporting information



Supporting Information

## Data Availability

The data that supports the findings of this study are available in the Supporting Information of this article.
