# Supplementary material for: Secretory Production of Heterologous Antimicrobial Peptides in Corynebacterium glutamicum
Source: Eng Life Sci. 2025 Feb 18;25(2):e70008. doi: 10.1002/elsc.70008 (PMC11835761; doi:10.1002/elsc.70008)
Supplement: Supplementary file 1 — Supporting Information [file ELSC-25-e70008-s001.pdf]

Supporting Information

Short Communication

Secretory production of heterologous antimicrobial peptides in *Corynebacterium glutamicum*

Material and methods

Bacterial strains, media and growth conditions

All experiments were performed with the *C. glutamicum* ATCC 13032 strain ***(1)***. *C. glutamicum* pre-cultures were cultivated at 30 °C in LB medium (1% (w/v) tryptone, 0.5% (w/v) yeast extract, 1% (w/v) NaCl) for Prsoriasin-fusion production or BHI medium (3.7% (w/v) BHI) for improved LCI-fusion production. In both cases, the medium contained 1% (w/v) glucose and 50 µg/ml kanamycin for maintenance of plasmid pEKEx2 and derivatives. *E. coli* strain DH5α ***(2)***, used for molecular cloning, were cultivated at 37 °C in LB medium containing 50 µg/ml kanamycin. Transformation was carried out using chemically competent cells for *E. coli* ***(3)*** and electrocompetent *C. glutamicum* cells ***(4)***.

Generation of AMP constructs

The constructs 17xHelix-TEV-AMP were cloned in the vector pEKEx2 containing the signal peptide NprE *via* phosphorothioate-based ligase-independent gene cloning (PLICing; ***(5)***). The Strep-taq II sequence was introduced by PCR primers and fused to the 5'-end of the 17xHelix. The inserts containing 17xHelix-TEV-AMP sequences ***(6, 7)*** were amplified using phosphorothioated primers ins_fwd (5'-agccatccgcagTTCGAAAAGGCAGAAGCAGCAGCAAAAGAAGCC-3') and ins_rev (5'-tcaatctctctgGCTTTGTTAGCAGCCGGATCTCAGTG-3'). The vector backbone harboring the NprE signal sequence was amplified with vb_fwd (5'-cagagagattgaATTCACTGGCCGTCGTTTTACAGC-3') and vb_rev

(5’-ctgcggatggctCCATGCAGAGAATTCAGCAGCCTGAACACCTGG-3’). Template DNA was digested with 20 U DpnI over 16 h at 37 °C and purified (QIAquick PCR Purification Kit, QIAGEN, Hilden, Germany) prior to hybridization of 0.004 µM vector backbone and 0.2 µM insert. The hybridized construct was amplified using *E. coli* DH5α with subsequent sequencing (Eurofins Genomics GmbH, Ebersberg, Germany) using Ptac sequencing primer (5’-ATCATCGGCTCGTATAATGTGTGG-3’).

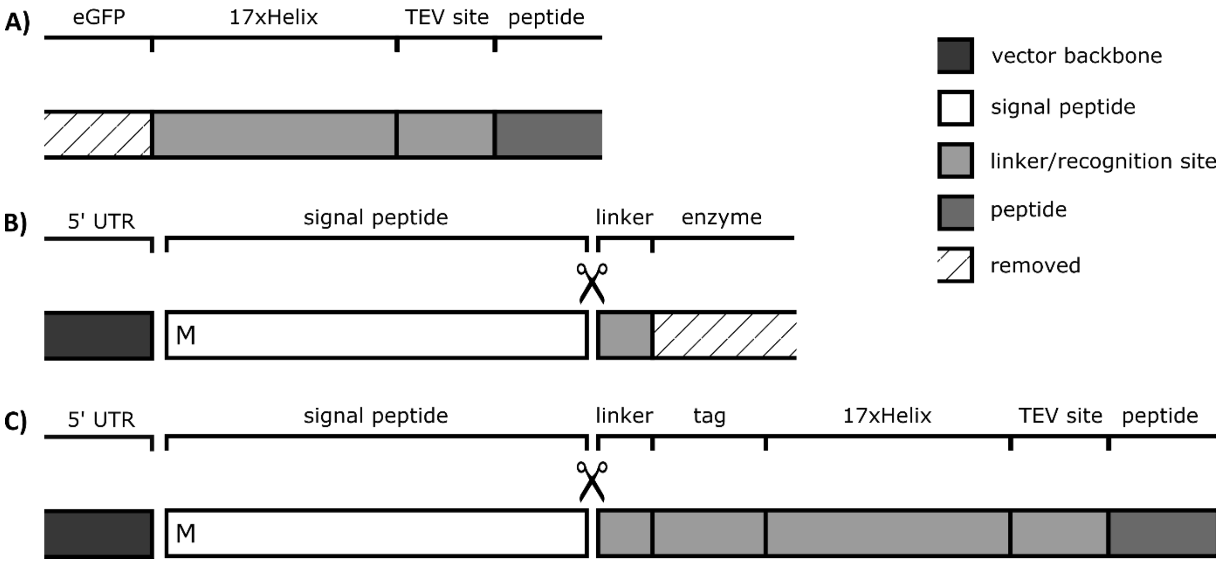

Figure S1: Genetic *amp* constructs. A) Starting construct pET28a-eGFP-17xHelix-TEV-AMP for insert generation, which contains a fluorescent protein (eGFP), a spacer (17xHelix), a TEV cleavage site (TEV site), and the AMP (peptide). B) Starting construct pEKEx2-NprE-enzyme for vector backbone generation, which contains a signal peptide, a linker and an enzyme. C) Final AMP construct with signal peptide linked (linker) to a Strep-tag II (tag), a spacer (17xHelix), a TEV cleavage site (TEV site), and the peptide. The translation start (M), the signal peptide cleavage site (scissor) are indicated. The vector backbone is indicated by the 5’ untranslated region (5’UTR).

Table S1: Sequence of constructs.

The 17xHelix-TEV-LCI insert and pEKEx2-NprE vector backbone were amplified with phosphorothioated (PTO) primers (ins_fwd: agc cat ccg cag TTC GAA AAG GCA GAA GCA GCA GCA AAA GAA GCC, ins_rev: tca atc tct ctg GCT TTG TTA GCA GCC GGA TCT CAG TG, vb_fwd: cag aga gat tga ATT CAC TGG CCG TCG TTT TAC AGC, vb_rev: ctg cgg atg gct CCA TGC AGA GAA TTC AGC AGC CTG AAC ACC TGG), which included the Strep-tag II sequence.

Part	Sequence	Reference
Signal peptide	MGLGKKLSVAVAASFMSLSISLPGVQA	***(8)***
Linker	AEF	-

Strep-tag II	SAWSHPQFEK	***(9)***
17xHelix	AEAAAKEAAAKEAAAKA	***(10)***
TEV cleavage site	ENLYFQG	***(11)***
LCI	AIKLVQSPNGNFAASFVLDGTKWIFKSKYYDSSKGYWV GIYEVWDRK	***(12)***
Psoriasin	MSNTQAERSIIGMIDMFHKYTRRDDKIDKPSLLTMMKE NFPNFLSACDKKGTNYLADVFEKKDKNEDKKIDFSEFLSL LGDIATDYHKQSHGAAPCSGGSQ	***(13)***

AMP production

For *C. glutamicum* AMP expression cultures, pre-cultures were used for inoculation of LB medium and subsequent incubating at 30 °C with shaking at 250 rpm. For optimized production of LCI, BHI medium was utilized and cultured at 25 °C under the same shaking conditions. For AMP production in deepwell plates, *C. glutamicum* was cultivated in 800 µL LB medium at 25°C, 900 rpm and a humidity of 70%. When the OD_{600} reached between 0.2 and 0.6, induction was carried out using IPTG to a final concentration of 0.1 mM. The LB culture continued overnight, while the BHI culture was maintained for a duration of 40 hours. Following cultivation, the cells were pelleted by centrifugation, allowing for the collection of the AMP-rich supernatant for further use in downstream applications.

Quantification of produced AMP constructs

For the quantification of produced LCI a Tricine-SDS-gel was casted according the published protocol by Schägger *et al.* ***(14)***. The supernatants and lysozyme gradient samples were mixed with 4x SDS loading dye and incubated at 98°C for 10 min. Samples were loaded onto the gel and separated. Afterwards, the gel was stained with Coomassie solution or Western blot followed by diaminobenzidine staining was performed. AMP concentration was determined by optical comparison of lysozyme gradient with corresponding AMP intensity using ImageJ ***(15)***. The Experion system, which is based on capillary gel electrophoresis (BioRad, Hercules, California, USA), was used for the

quantification of psoriasin. After adding an internal lysozyme standard (final concentration of 100 ng/µl), the psoriasin-rich supernatants were prepared according to the manufacturer's instructions, measured and analyzed with the Experion software.

Detection of AMP binding to MTPs

The binding assay developed by Rubsam *et al.,* ***(6, 7)*** was adapted. The AMP containing supernatant was loaded onto PS-MTP wells (Greiner Bio-One, 96-well, F-Bottom, clear) and incubated (100 µL, 10 min, RT) in PBS buffer (6.73 g/L NaCl, 2.85 g/L Na_2HPO_4, 0.54 g/L KH_2PO_4, 1:100 dilution). Two washing steps (110 µl, 5 min, 600 rpm) were performed with PBST (0.05% (v/v) Tween-20 in PBS) to remove unbound molecules. Afterwards, Strep-Tactin-HRP conjugate (iba, Goettingen, Germany) was added (42 µg/L in PBS, 1h, 600 rpm) to bind to the Strep-tag II of the corresponding AMP. Unbound conjugates were removed by washing twice (110 µl PBST, 5 min, 600 rpm). 100 µL of the 1-Step Ultra TMB Elisa solution (Thermo Fischer Scientific, Waltham, USA) was added to detect bound AMPs indirectly *via* HRP activity. The reaction was stopped with 2 M sulfuric acid and was measured at 450 nm (Tecan Sunrise, TECAN, Männedorf, Switzerland).

Influence of AMPs on *C. glutamicum* growth

To investigate the influence of antimicrobial peptides (AMPs) on the growth of *C. glutamicum*, AMP-rich supernatants were concentrated tenfold using centrifugal filters with a 3 kDa cutoff (Amicon, Sigma-Aldrich, Missouri, USA). The concentrated supernatants were then added to LB medium, which was inoculated with 1/30 of a pre-culture of *C. glutamicum* in MTPs. Different concentrations of AMPs were introduced into the medium to assess their effects on bacterial growth. As a positive control for antimicrobial effects, Ampicillin was used at a final concentration of 100 µg/ml. Cultivation was carried out at 30 °C, and the optical density was measured at 600 nm every 15 minutes after prior shaking to ensure homogeneity in the culture.

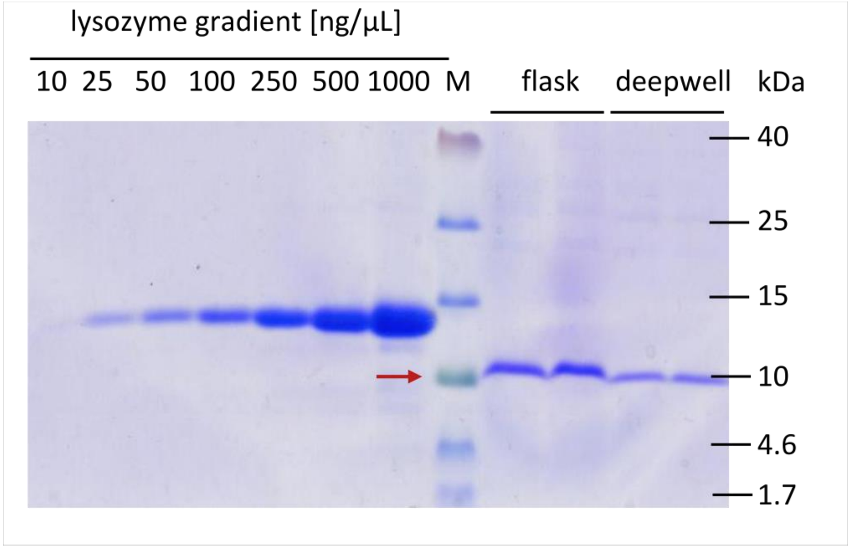

Figure S2: LCI quantification for flask and deepwell production. Tricine-SDS-gel of LCI produced in flask and deepwell plate. The expected band is indicated by a red arrow. Different concentrations of lysozyme served as concentration gradient. Molecular weight was estimated by Spectra Multicolor Low Range Protein Ladder (Thermo Fischer Scientific, indicated with M).

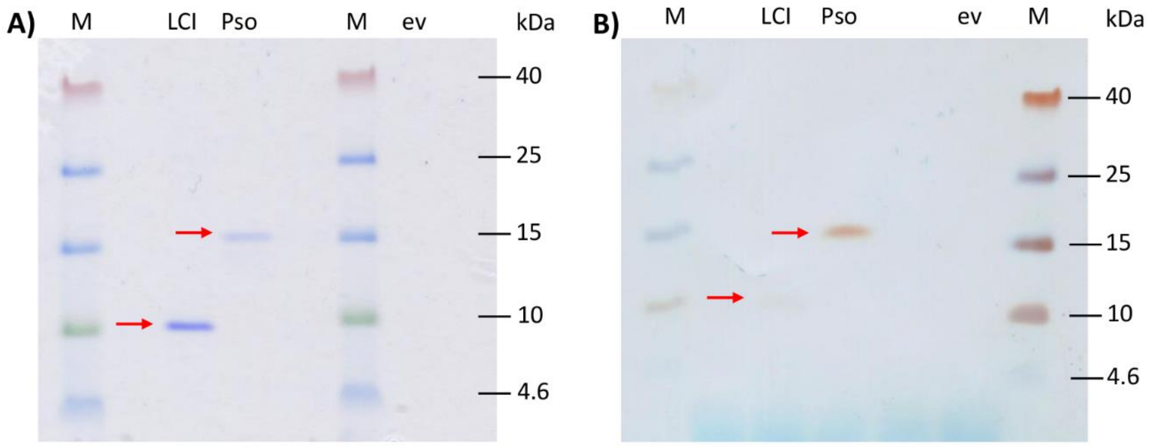

Figure S3: Detection of produced AMPs. LCI, Psoriasin (Pso) and an empty vector control (ev) were expressed overnight in flask (LB-medium, 30°C, 250 rpm). The supernatant of the expression culture was obtained by centrifugation (4°C, 3200 g, 20 min) and analyzed on a Tricine-sodium dodecyl sulfate-gel (Tricine-SDS-gel) according to Schägger et al. (Schägger and von Jagow, 1987). Bands (indicated by red arrows) were visualized by A) Coomassie staining and B) 3,3'-Diaminobenzidine stained Western

Blot. Molecular weight was estimated by Spectra Multicolor Low Range Protein Ladder (Thermo Fischer Scientific, indicated with M).

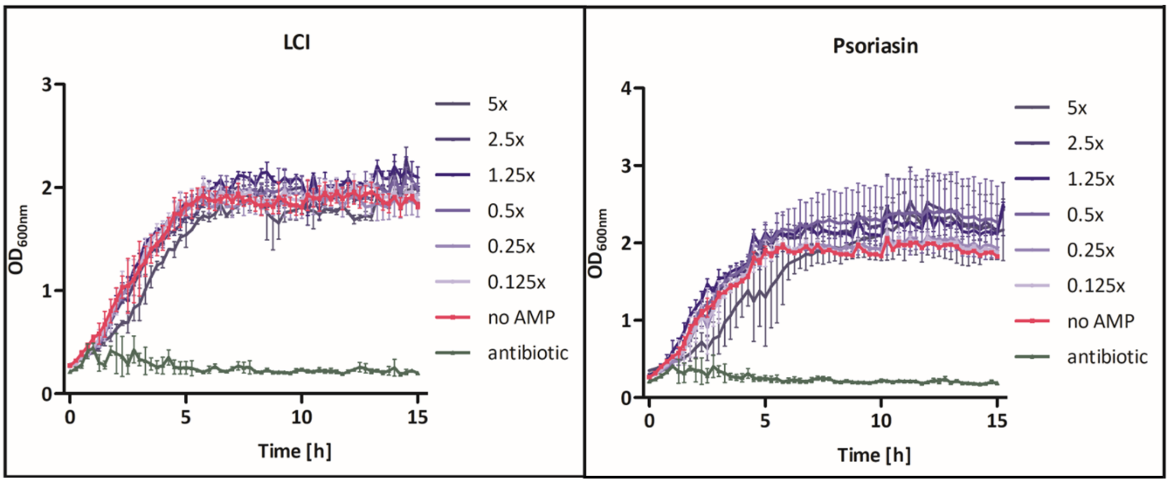

Figure S4: Analyzing the AMP's influence on *C. glutamicum* growth. The AMP-rich supernatant from an expression culture was initially concentrated tenfold using centrifugal filters with a 3 kDa cutoff. This concentrate was then diluted in LB medium within a MTP, resulting in concentrations ranging from 5x to 0.125x of the original supernatant for analysis. Each well was inoculated with 1/30 of a *C. glutamicum* pre-culture, and cultures were incubated at 30°C for 15 hours. Growth was monitored every 15 minutes by measuring optical density at 600 nm. Ampicillin (final concentration of 100 µg/ml) served as a positive control for antimicrobial efficacy, while cultures without AMP or antibiotic acted as negative controls.

References

1. Kinoshita, S., Udaka, S. and Shimono, M. (1957) Studies on the amino acid fermentation Part I. Production of L-glutamic acid by various microorganisms. The Journal of general and applied microbiology 3, 193-205.
2. Woodcock, D., Crowther, P., Doherty, J., Jefferson, S., DeCruz, E., Noyer-Weidner, M., Smith, S., Michael, M. and Graham, M. (1989) Quantitative evaluation of Escherichia coli host strains for tolerance to cytosine methylation in plasmid and phage recombinants. Nucleic acids research 17, 3469-3478.
3. Maniatis, T. (1982) Molecular cloning. A laboratory manual.
4. Lessard, P. (2002) Electrocompetent *C. glutamicum* and *Rhodococcus* sp. B264-1, vol. 2024.
5. Blanusa, M., Schenk, A., Sadeghi, H., Marienhagen, J. and Schwaneberg, U. (2010) Phosphorothioate-based ligase-independent gene cloning (PLICing): An enzyme-free and sequence-independent cloning method. Analytical Biochemistry 406, 141-146.
6. Rübsam, K., Stomps, B., Böker, A., Jakob, F. and Schwaneberg, U. (2017) Anchor peptides: A green and versatile method for polypropylene functionalization. Polymer 116, 124-132.
7. Rübsam, K., Weber, L., Jakob, F. and Schwaneberg, U. (2018) Directed evolution of polypropylene and polystyrene binding peptides. Biotechnology and Bioengineering 115, 321-330.
8. Tjalsma, H., Bolhuis, A., Jongbloed, J. D. H., Bron, S. and van Dijl, J. M. (2000) Signal peptide-dependent protein transport in Bacillus subtilis: a genome-based survey of the secretome. Microbiol. Mol. Biol. Rev. 64, 515-547.
9. Schmidt, T. G. M. and Skerra, A. (2007) The Strep-tag system for one-step purification and high-affinity detection or capturing of proteins. Nature Protocols 2, 1528-1535.

10. Arai, R., Ueda, H., Kitayama, A., Kamiya, N. and Nagamune, T. (2001) Design of the linkers which effectively separate domains of a bifunctional fusion protein. Protein Engineering, Design and Selection 14, 529-532.
11. Parks, T. D., Leuther, K. K., Howard, E. D., Johnston, S. A. and Dougherty, W. G. (1994) Release of Proteins and Peptides from Fusion Proteins Using a Recombinant Plant Virus Proteinase. Analytical Biochemistry 216, 413-417.
12. Gong, W., Wang, J., Chen, Z., Xia, B. and Lu, G. (2011) Solution Structure of LCI, a Novel Antimicrobial Peptide from *Bacillus subtilis*. Biochemistry 50, 3621-3627.
13. Gläser, R., Harder, J., Lange, H., Bartels, J., Christophers, E. and Schröder, J.-M. (2004) Antimicrobial psoriasin (S100A7) protects human skin from Escherichia coli infection. Nat. Immunol. 6, 57-64.
14. Schägger, H. and Von Jagow, G. (1987) Tricine-sodium dodecyl sulfate-polyacrylamide gel electrophoresis for the separation of proteins in the range from 1 to 100 kDa. Analytical biochemistry 166, 368-379.
15. Schneider, C. A., Rasband, W. S. and Eliceiri, K. W. (2012) NIH Image to ImageJ: 25 years of image analysis. Nature methods 9, 671-675.
